# Supplementary material for: MQuad enables clonal substructure discovery using single cell mitochondrial variants
Source: Nat Commun. 2022 Mar 8;13:1205. doi: 10.1038/s41467-022-28845-0 (PMC8904442; doi:10.1038/s41467-022-28845-0)
Supplement: Supplementary file 1 — Supplementary information [file 41467_2022_28845_MOESM1_ESM.pdf]

Supplementary file for "MQuad enables clonal substructure discovery using single cell mitochondrial variants"

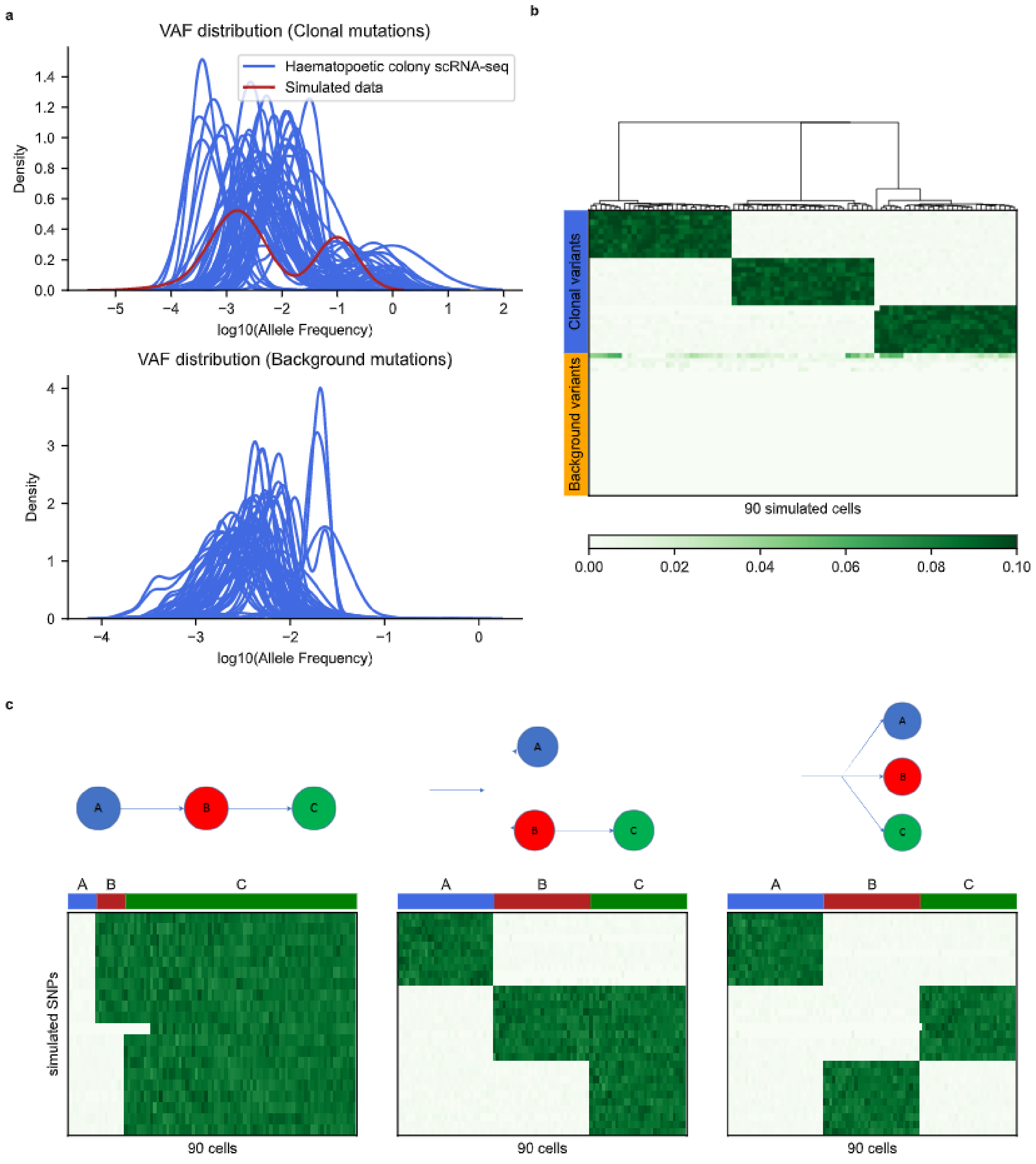

Supplementary Fig. 1: Additional data on simulation settings.

a, VAF distribution of clonal mutations (top) and background mutations (bottom) found in hematopoietic colony scRNA-seq dataset. Red line indicates the distribution of VAF of simulated clonal variants.

b, VAF heatmap with hierarchical clustering of 30 simulated clonal variants and 30 random background mutations. Rows are variants, columns are cells. Simulation settings shown here are default: 10 informative variants in each clone, 10% VAF, equal clone sizes, fully branched tree.

c, Tree topologies (top) and VAF heatmap of clonal variants reflecting the corresponding clonal dynamics (bottom) .

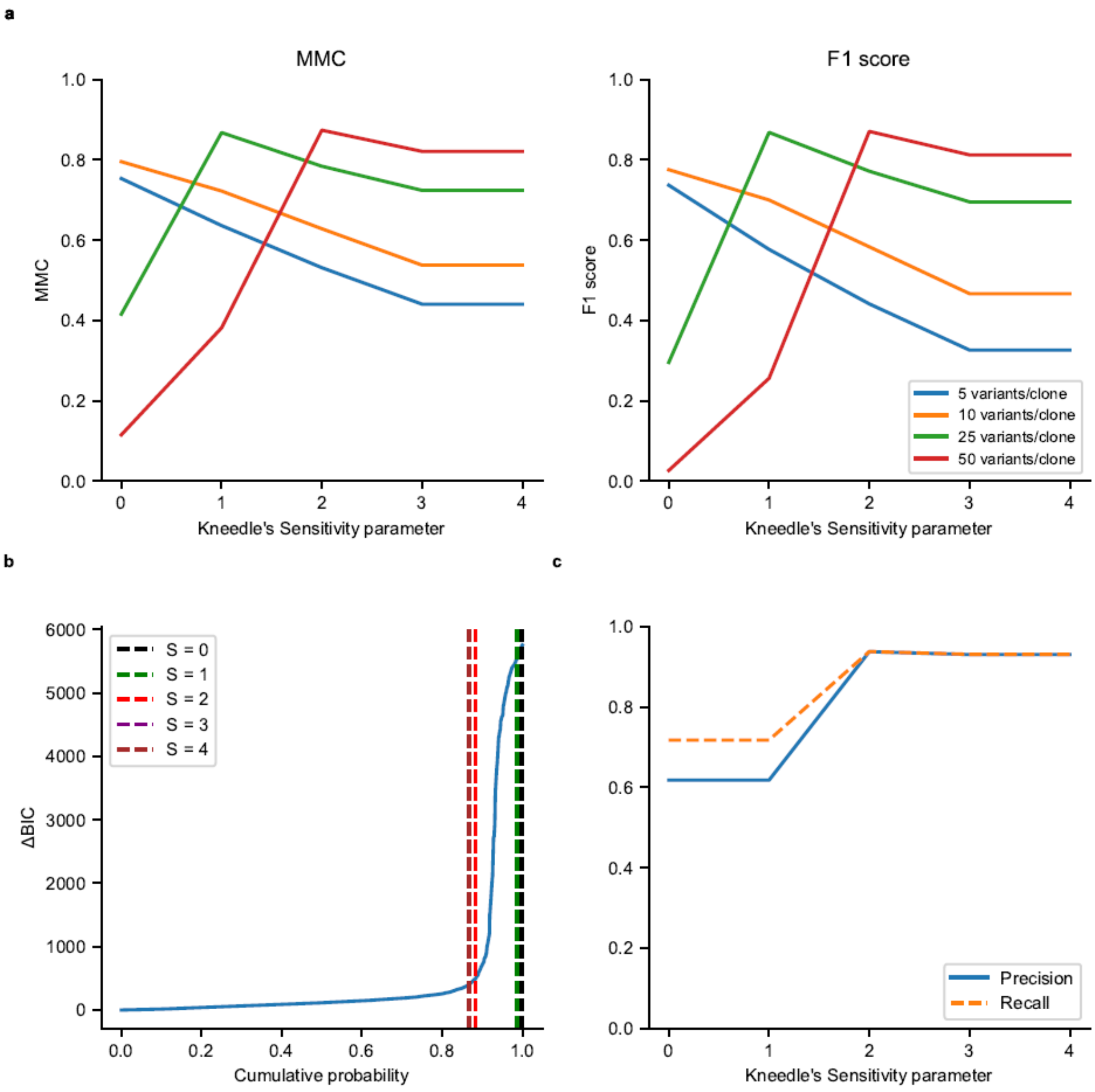

Supplementary Fig. 2: Effect of varying the sensitivity parameter in Kneedle.

a, Change in MMC (left) and F1 score (right) with regard to variant detection when varying the sensitivity parameter in Kneedle on simulated datasets.

b, Cumulative probability of ΔBIC in Kim ccRCC dataset with different Kneedle cutoffs. S represents sensitivity. N.B. the line for S=3 is covered by the line for S=4.

c, Change in precision and recall for clonal assignment in Kim ccRCC dataset, using variants identified from varying Kneedle cutoffs.

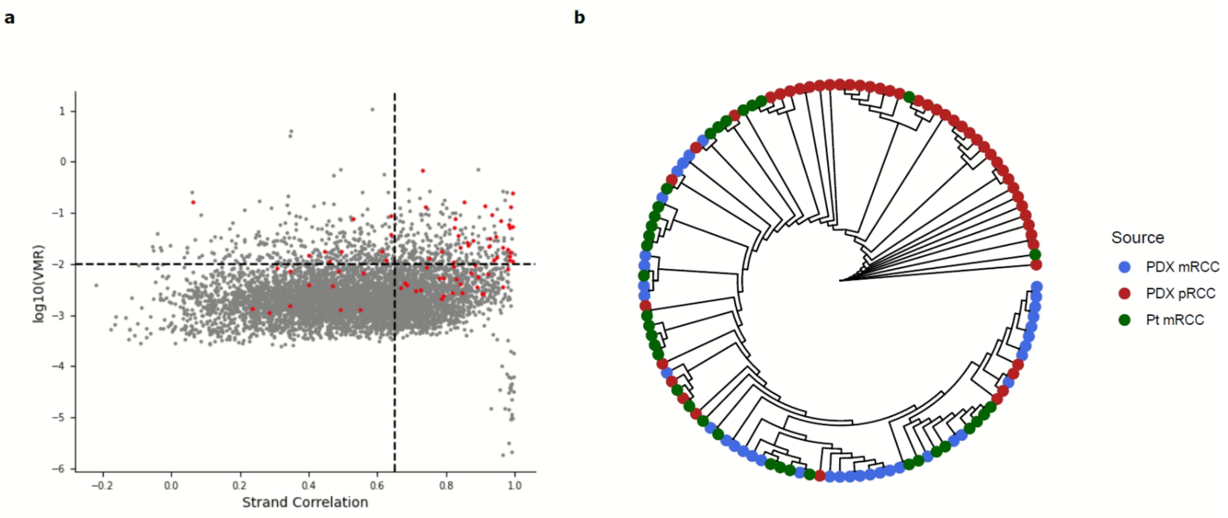

Supplementary Fig. 3: Extended analysis on Kim ccRCC dataset.

a, Output from mgatk showing log(VMR) against strand correlation. Each dot is a variant, variants detected by MQuad are highlighted in red. Dotted lines are default thresholds for informativeness determination.

b, Phylogenetic tree inferred by SCITE use MQuad variants as input. N.B., SCITE takes an input of presence and absence of alternative allele, hence we set a threshold 0.9 > VAF >0.1 as heterozygous alternate, VAF ≧ 0.9 as homozygous alternate, and VAF ≦ 0.1 as homozygous reference.

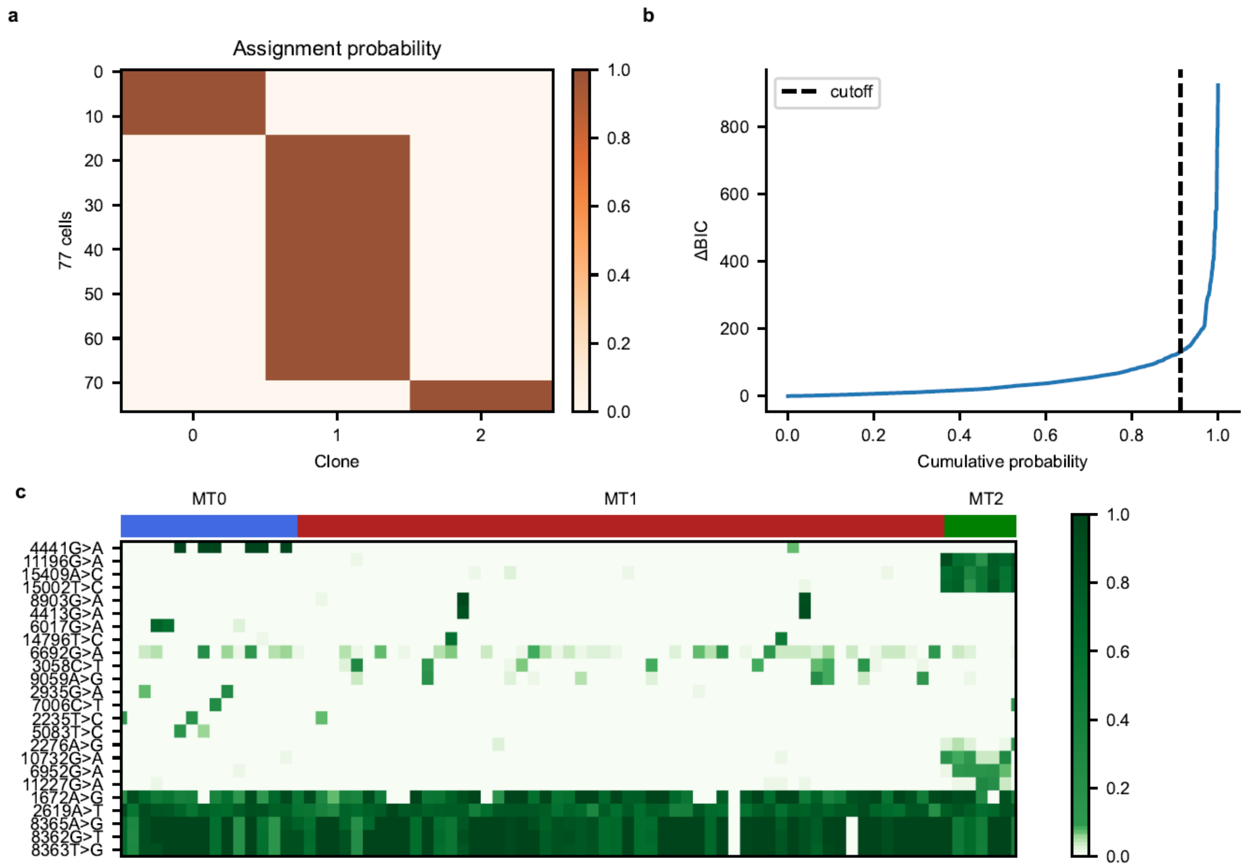

Supplementary Fig. 4: Extended analysis on joxm fibroblast dataset.

a, Clone assignment heatmap from vireoSNP. Rows are cells and columns are clones.

b, Cumulative probability of ΔBIC with Kneedle cutoff shown.

c, Allele frequency heatmap for informative variants detected.

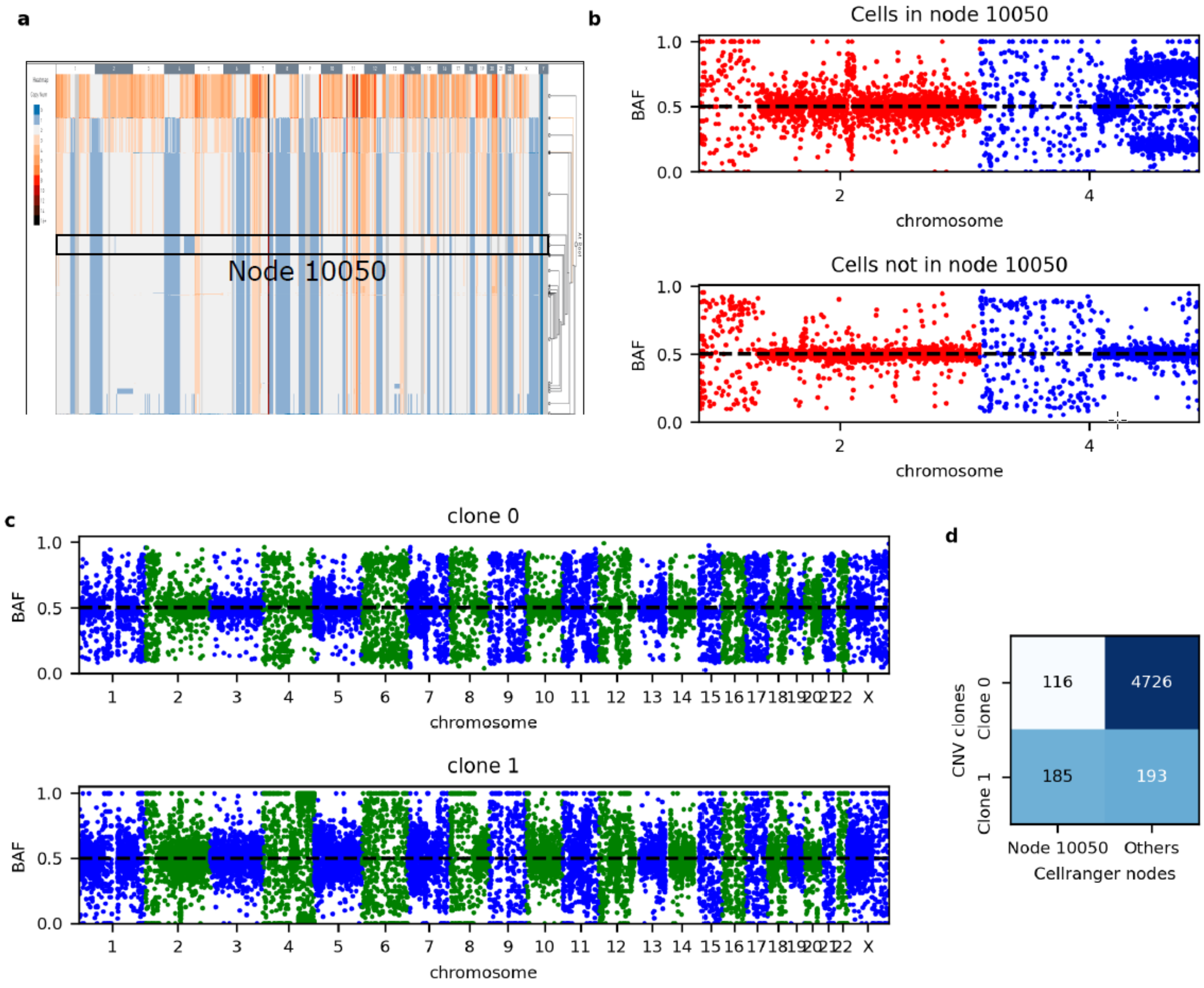

Supplementary Fig. 5: Averaged B-allele frequency for CNV clones identified by VireoSNP binomial mixture model.

a, Cellranger CNV profile of MKN-45 cell line. Node 10050 highlighted.

b, Averaged B-allele frequency (BAF) on chr2 and 4 for cells in node 10050 (top) and cells not in the node (bottom).

c, Averaged BAF on all chromosomes for CNV clone 0 (top) and clone 1 (bottom) that are identified with BAF on chr2 and chr4 (see Methods).

d, Confusion matrix between assigned CNV clones and original Cellranger nodes.

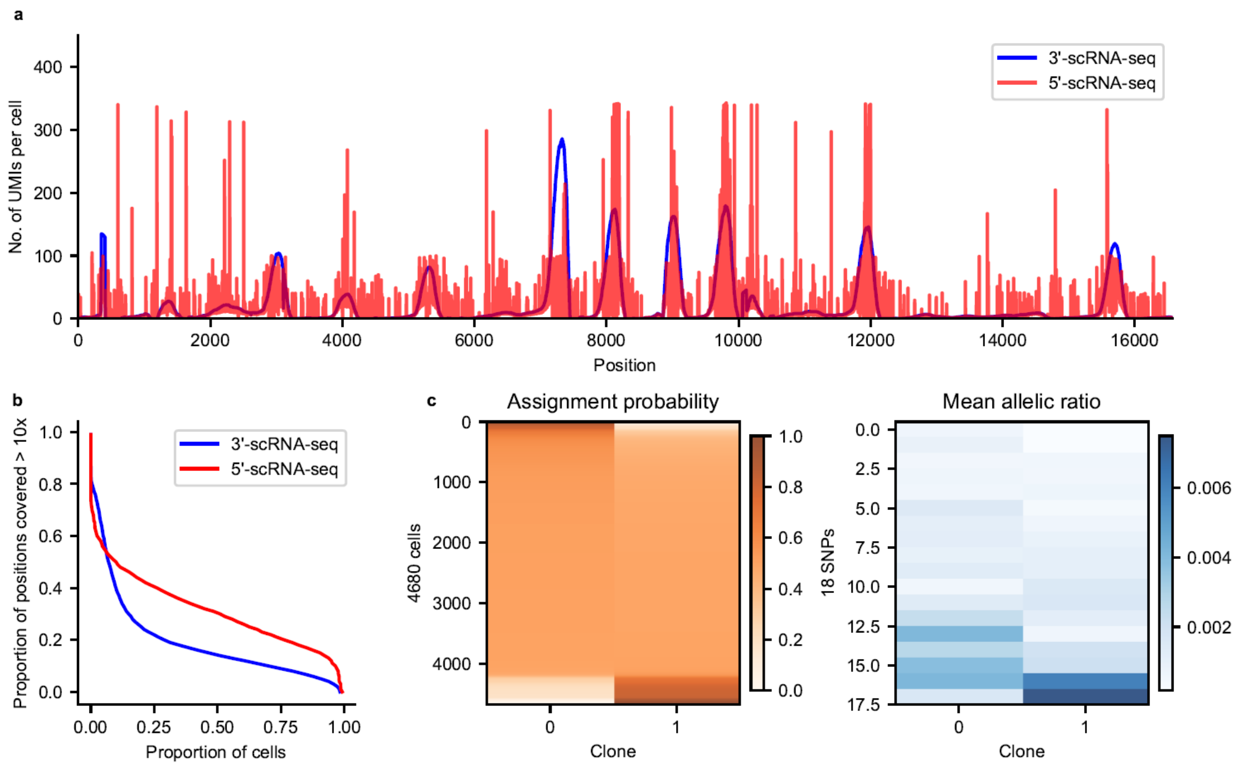

Supplementary Fig. 6: Additional analysis on melanoma 10x 5' scRNA-seq.

a, Comparison of number of UMIs per cell in 3' vs 5'-scRNA-seq

b, Comparison of proportion of cells with proportion of positions covered more than 10 times in 3' vs 5'-scRNA-seq.

c, Clone assignment heatmap for 5'-scRNA-seq dataset.

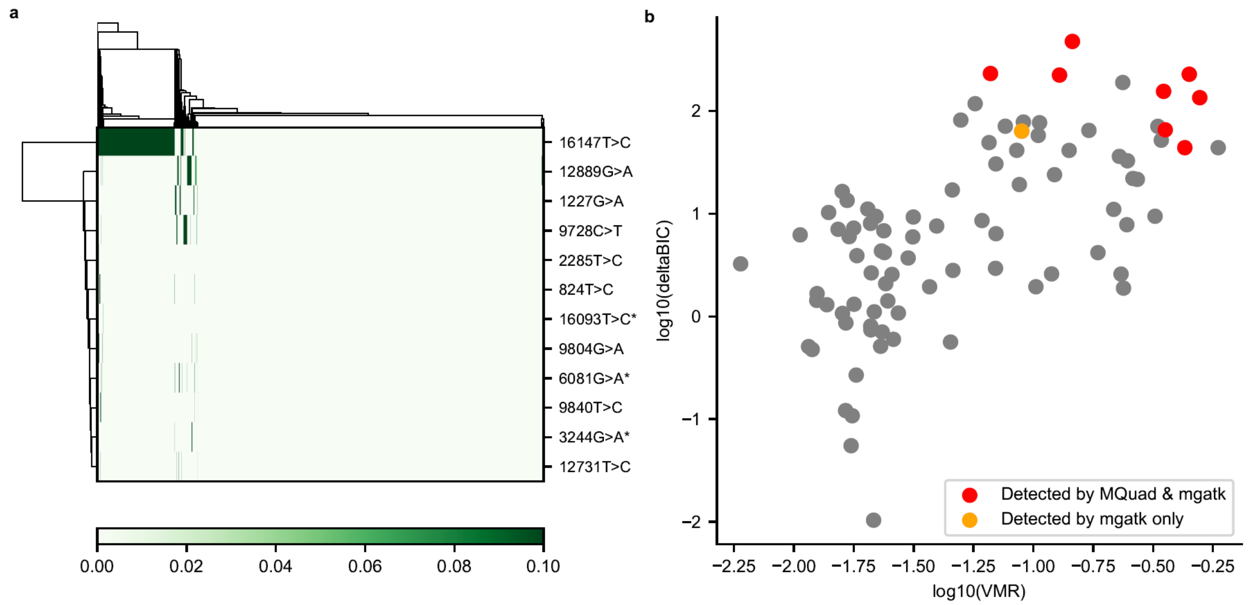

Supplementary Fig. 7: Mitochondrial variants detected in CRC mtscATAC-seq.

a, Allele frequency heatmap of variants detected by mgatk and MQuad. Asterisk denotes variants that are only detected by mgatk.

b, Scatter plot of variants with log10(deltaBIC) against log10(VMR). Variants detected by both tools are in red. 6081G>A (orange) had a positive deltaBIC but not high enough to make the knee point. 3244G>A and 16093T>C were not detected by MQuad due to too low of a signal.

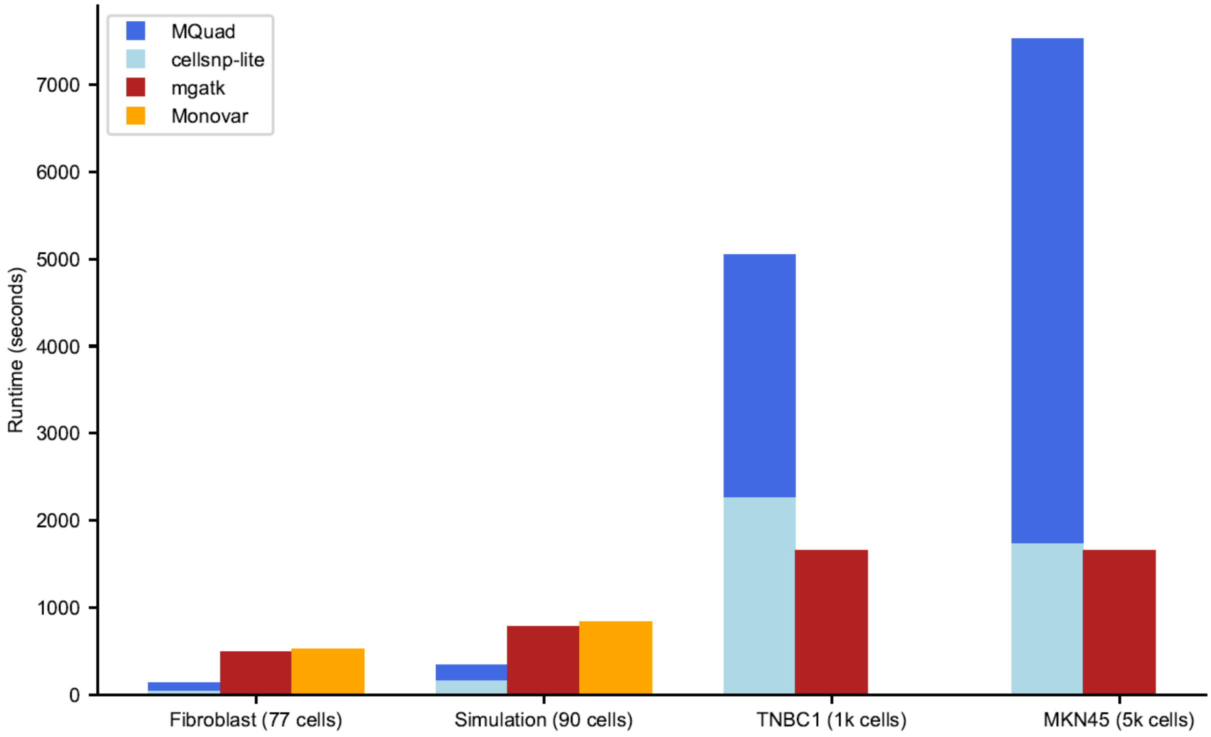

Supplementary Fig. 8: Runtime comparison on datasets of various sizes.

All tools are benchmarked on Intel Xeon Gold 6238R CPU with 10 threads.

N.B. Monovar cannot be run on 10x datasets as it takes a list of bam files as input and cannot demultiplex 10x bam files with barcodes.

Sequencing platform	Dataset	# cells	Mean MT reads	Coverage saturation	# informative variants	Assignable proportion	n_clones
Smart-seq2	Kim ccRCC	121	234,294	Good	146	100%	3
	Fibroblast	77	27,334	Good	24	100%	3
mtscATAC-seq	CRC	3,559	28,273 (1,774*)	Moderate	9	25% (52% for high quality cells**)	3
10x CNV	MKN-45	5,220	8,020	Good	24	93%	5
			4,010	Moderate	15	88%	5
			2,005	Moderate	15	81%	5
10x 3' scRNA-seq	TNBC1	1,097	36,381	Moderate	2	96%	2
	TNBC2	1,034	22,705	Poor	3	0%	NA
	TNBC5	3,225	35,337	Poor	17	5%	2
10x 5' seq	Melanoma	4,680	4,420	Poor	18	8%	2

Supplementary Table 1: Summary of all datasets used. Mean MT reads is estimated by dividing the total number of reads in the big bam file (samtools idxstats) by the number of cell barcodes used.

* The number of reads per cell decreases drastically after filtering low quality reads with default cellsnp-lite parameters (samtools view -q 20 -m 20 - F 1796).

** The default 3,559 cells are called by CellRanger and the high-quality cell barcodes (1,505 cells) are defined from the original paper. The relatively low assignability is caused by the majority of cells having nearly no coverage on those clonal variants; if only the high quality cells are considered, the assignability is improved.

Algorithm 1: Expectation Maximization

Input: AD, DP, K=2

Output: $\pi_k, \theta_k, (k = 0, .., K-1)$

for *each k* **do**

  Randomly Initialize π_k, θ_k,

  $s.t. \sum_k \pi_k = 1;\ 0 \leq \theta_k \leq 1$

end

while *Not Convergence* **do**

  # E-step:

  for *each k* **do**

    for *j=1:M* **do**

      $$\bar{\gamma}_k^{(j)} := \frac{\theta_k^{AD_j}(1-\theta_k)^{(DP_j - AD_j)} \cdot \pi_k}{\sum_k \theta_k^{AD_j}(1-\theta_k)^{(DP_j - AD_j)} \cdot \pi_k}$$

    end

  end

  # M-step:

  for *each k* **do**

    $$\pi_k = \frac{\sum_{j=1}^{M} \bar{\gamma}_k^{(j)}}{N};\ \theta_k = \frac{\sum_{j=1}^{M} AD_j \bar{\gamma}_k^{(j)}}{\sum_{j=1}^{M} DP_j \bar{\gamma}_k^{(j)}}$$

  end

end

Supplementary Algorithm 1: Expectation Maximization
