# Supplementary material for: MQuad enables clonal substructure discovery using single cell mitochondrial variants
Source: Nat Commun. 2022 Mar 8;13:1205. doi: 10.1038/s41467-022-28845-0 (PMC8904442; doi:10.1038/s41467-022-28845-0)
Supplement: Supplementary file 2 — Reporting Summary [file 41467_2022_28845_MOESM2_ESM.pdf]

nature portfolio

Corresponding author(s): Yuanhua Huang & Joshua W.K. Ho

Last updated by author(s): Feb 13, 2022

Reporting Summary

Nature Portfolio wishes to improve the reproducibility of the work that we publish. This form provides structure for consistency and transparency in reporting. For further information on Nature Portfolio policies, see our Editorial Policies and the Editorial Policy Checklist.

Statistics

For all statistical analyses, confirm that the following items are present in the figure legend, table legend, main text, or Methods section.

n/a	Confirmed	
☐	☒	The exact sample size (*n*) for each experimental group/condition, given as a discrete number and unit of measurement
☐	☒	A statement on whether measurements were taken from distinct samples or whether the same sample was measured repeatedly
☐	☒	The statistical test(s) used AND whether they are one- or two-sided<br>*Only common tests should be described solely by name; describe more complex techniques in the Methods section.*
☐	☒	A description of all covariates tested
☐	☒	A description of any assumptions or corrections, such as tests of normality and adjustment for multiple comparisons
☐	☒	A full description of the statistical parameters including central tendency (e.g. means) or other basic estimates (e.g. regression coefficient) AND variation (e.g. standard deviation) or associated estimates of uncertainty (e.g. confidence intervals)
☐	☒	For null hypothesis testing, the test statistic (e.g. *F*, *t*, *r*) with confidence intervals, effect sizes, degrees of freedom and *P* value noted<br>*Give P values as exact values whenever suitable.*
☐	☒	For Bayesian analysis, information on the choice of priors and Markov chain Monte Carlo settings
☐	☒	For hierarchical and complex designs, identification of the appropriate level for tests and full reporting of outcomes
☐	☒	Estimates of effect sizes (e.g. Cohen's *d*, Pearson's *r*), indicating how they were calculated

Our web collection on statistics for biologists contains articles on many of the points above.

Software and code

Policy information about availability of computer code

Data collection	Sratoolkit (2.9.1) was used to download publicly available data from GEO. NEAT-genReads (3.0) was used to simulate reads used in Fig. 1.
Data analysis	Software used for data analysis includes Python (3.8.8), R (3.6.3), cellSNP-lite (1.2.1), MQuad (0.1.6), vireoSNP (0.5.3), kneed (0.7.0), STAR (2.7.2a), Picard (2.18.9), edgeR (3.28.1), limma (3.34.2), IHW (1.14.0), Seurat (3.2.1), SomatoSim(1.0.0), SCITE, Monovar.

For manuscripts utilizing custom algorithms or software that are central to the research but not yet described in published literature, software must be made available to editors and reviewers. We strongly encourage code deposition in a community repository (e.g. GitHub). See the Nature Portfolio guidelines for submitting code & software for further information.

Data

Policy information about availability of data

All manuscripts must include a data availability statement. This statement should provide the following information, where applicable:

- Accession codes, unique identifiers, or web links for publicly available datasets
- A description of any restrictions on data availability
- For clinical datasets or third party data, please ensure that the statement adheres to our policy

All datasets used are all publicly available, through the NCBI Gene Expression Omnibus (GEO) portal or ArrayExpress database at EMBL-EBI, with accession numbers GSE73121 (Kim dataset), GSE148673 (TNBC datasets), GSE148509 (CRC dataset), E-MTAB-7167 (Fibroblast dataset).
MKN-45 scDNA-seq (https://www.10xgenomics.com/resources/datasets/5-k-mkn-45-gastric-cancer-cell-line-750-k-read-pairs-per-cell-1-standard-1-1-0)
and the melanoma 5’ scRNA-seq datasets (https://support.10xgenomics.com/single-cell-vdj/datasets/6.0.1/SC5v2_Melanoma_5Kcells_Connect_single_channel_SC5v2_Melanoma_5Kcells_Connect_single_channel), in bam format, are both downloaded from 10X Genomics website.

March 2021

Field-specific reporting

Please select the one below that is the best fit for your research. If you are not sure, read the appropriate sections before making your selection.

☒ Life sciences ☐ Behavioural & social sciences ☐ Ecological, evolutionary & environmental sciences

For a reference copy of the document with all sections, see nature.com/documents/nr-reporting-summary-flat.pdf

Life sciences study design

All studies must disclose on these points even when the disclosure is negative.

Sample size	No sample size calculation was performed. Sample size was determined by the data downloaded from public databases. No new experimental data was generated in this study.
Data exclusions	No data is excluded
Replication	As all datasets used are downloaded from public databases, experimental replication is not applicable.
Randomization	N/A, each dataset contains samples from one individual/cell line
Blinding	N/A, each dataset contains samples from one individual/cell line

Reporting for specific materials, systems and methods

We require information from authors about some types of materials, experimental systems and methods used in many studies. Here, indicate whether each material, system or method listed is relevant to your study. If you are not sure if a list item applies to your research, read the appropriate section before selecting a response.

Materials & experimental systems

n/a	Involved in the study	
☒	☐	Antibodies
☒	☐	Eukaryotic cell lines
☒	☐	Palaeontology and archaeology
☒	☐	Animals and other organisms
☒	☐	Human research participants
☒	☐	Clinical data
☒	☐	Dual use research of concern

Methods

n/a	Involved in the study	
☒	☐	ChIP-seq
☒	☐	Flow cytometry
☒	☐	MRI-based neuroimaging
